# Imprinted Genes and the Environment: Links to the Toxic Metals Arsenic, Cadmium and Lead

**DOI:** 10.3390/genes5020477

**Published:** 2014-06-11

**Authors:** Lisa Smeester, Andrew E. Yosim, Monica D. Nye, Cathrine Hoyo, Susan K. Murphy, Rebecca C. Fry

**Affiliations:** 1 Department of Environmental Sciences and Engineering, Gillings School of Global Public Health, The University of North Carolina, 135 Dauer Drive, CB 7431, UNC, Chapel Hill, NC 27599, USA; E-Mails: smesta@unc.edu (L.S.); yosim@unc.edu (A.E.Y.); 2 Lineberger Comprehensive Cancer Center, The University of North Carolina, 450 West Street, CB 7295, UNC, Chapel Hill, NC 27599, USA; E-Mail: mnye@email.unc.edu; 3 Department of Obstetrics and Gynecology, Duke University Medical Center, B226 LSRC, Box 91012, Research Drive, Durham, NC 27708, USA; E-Mail: susan.murphy@duke.edu; 4 Department of Biological Sciences, Center for Human Health and Environment, Campus Box 7633, NC State University, Raleigh, NC 27695, USA; E-Mail: choyo@ncstate.edu; 5 Curriculum in Toxicology, The University of North Carolina, 135 Dauer Drive, CB 7431, UNC, Chapel Hill, NC 27599, USA

**Keywords:** imprinted genes, epigenetics, DNA methylation, TP53, aryl hydrocarbon receptor, systems biology, environmental health, toxic metals, epigenomics, environment

## Abstract

Imprinted genes defy rules of Mendelian genetics with their expression tied to the parent from whom each allele was inherited. They are known to play a role in various diseases/disorders including fetal growth disruption, lower birth weight, obesity, and cancer. There is increasing interest in understanding their influence on environmentally-induced disease. The environment can be thought of broadly as including chemicals present in air, water and soil, as well as food. According to the Agency for Toxic Substances and Disease Registry (ATSDR), some of the highest ranking environmental chemicals of concern include metals/metalloids such as arsenic, cadmium, and lead. The complex relationships between toxic metal exposure, imprinted gene regulation/expression and health outcomes are understudied. Herein we examine trends in imprinted gene biology, including an assessment of the imprinted genes and their known functional roles in the cell, particularly as they relate to toxic metals exposure and disease. The data highlight that many of the imprinted genes have known associations to developmental diseases and are enriched for their role in the TP53 and AhR pathways. Assessment of the promoter regions of the imprinted genes resulted in the identification of an enrichment of binding sites for two transcription factor families, namely the zinc finger family II and PLAG transcription factors. Taken together these data contribute insight into the complex relationships between toxic metals in the environment and imprinted gene biology.

## 1. Introduction

There is heightened interest in understanding the role of epigenetic mechanisms in cell signaling regulation and disease. This is particularly the case when attempting to discern the etiology of disease where a cause is hitherto unknown. Recent studies suggest that disease can be influenced by the environment via epigenetic mechanisms [1,2,3], a seemingly Lamarkian notion discordant with the tenets set forth by Mendel’s work. Yet the advent of modern epigenetics as a distinct field of study is much more storied than simply pitting Lamark’s theory of “soft inheritance” against Mendel’s firm genetic basis of heredity. While controversial to this day, some of the earliest experiments that suggested non-Mendelian inheritance, and possibly the first indication of parent-of-origin phenomena, came from Kammerer’s midwife toad experiments [4] which pointed to the ability of a modified environment to modulate heritable shifts in mating.

However, it was not until the early 1940s, the same time Huxley’s Modern Synthesis sought an interdisciplinary approach to inheritance and evolution [5], that the term epigenetics was first introduced by developmental biologist Conrad Waddington [6]. Describing mechanisms that determine cell fate and differentiation during development, Waddington’s epigenetics arose as a conceptual means to describe how a complex network of genes and gene-environment interactions brought about phenotype in an evolutionary context [7].

It was more recently though, with Holliday linking the term epigenetics with the ability of DNA methylation to modulate gene activity, that the definition of epigenetics began to shift to being inclusive of any mechanism with the ability to modulate gene activity without a change in DNA sequence [8]. These epigenetic modifications to an individual’s genome include, but are not limited to, three commonly studied mechanisms: DNA methylation, histone modification, and non-coding RNA expression [9].

While such marks are generally stable and heritable in nature, there is the potential for epigenetic modifications to be reversible, as seen with their innate reversibility during critical stages of fetal development, as epigenetic tags are added and removed [10,11,12]. Such changes to the epigenetic landscape are vital in the process of normal development [13], yet imprinted genes are protected from this process [14].

Changes to the epigenome can also be induced by the environment resulting in abnormal physiologic changes [3,15]. Stable epigenetic modifications are implicated in adult onset disease such as cancer, neurodevelopmental/neurodegenerative disorders, and autoimmune disorders [16] among others, and also have the potential to be trans-generational in nature [17,18]. However, it should be noted that the stability of such epigenetic alterations and their link to later life health outcomes is still under active debate [19,20].

Current research has shown that epigenetic modifications can be induced by exposure to environmental contaminants, such as toxic metals [21,22,23]. Such modifications have been linked to later life health outcomes including cancers, heart disease, kidney disease, and various neurological conditions [20,24,25]. While cadmium and lead are metals, arsenic is a metalloid, with shared properties of both metals and non-metals. For the purposes of this article, all three elements will hereby be referred to as “metals.” While these metals are among the most studied, many other metals have demonstrated toxicity and are associated with epigenetic alterations including nickel and chromium [21]. In addition, new research is currently investigating various under-studied metals for toxicity and associated epigenetic alterations including tungsten and cobalt [26,27].

While research continues into the stability of epigenetic alterations associated with environmental contaminants and their associations with negative health endpoints, many have theorized that such reversibility may provide the opportunity for therapeutic targets for disease prevention following environmental exposure [24,28].

### 1.1. What is Genomic Imprinting?

Evidence that parental genomes are not equivalent was first described in mouse models by both the Surani and McGrath groups in the early 1980s [29,30]. The researchers attempted to generate viable embryos using only maternal or paternal chromosomes. They found that normal development required genetic material from both parents; maternal and paternal genomes were not interchangeable, indicating the first experiments to demonstrate mammalian imprinting. While the exact mechanism of these imprinting phenomena was unknown at that time, they hypothesized a process that operated pre-fertilization, yet impacted post-fertilization expression. To date, researchers have used a variety of strategies including genome-wide studies, gene-specific experiments, and transcriptome analysis to determine which human or mammalian genes are imprinted [31,32,33]. Imprinted genes are vulnerable to genetic and epigenetic perturbation and have been tied to adverse health outcomes. As imprinted genes are monoallelically expressed with one of the copies of the gene silenced in a parent-of-origin dependent manner, only one copy is functional. As a result, mutations or epigenetic alterations on one allele that would normally have minimal impact for a biallelically expressed gene may lead to detrimental consequences for an imprinted gene.

As it is a critical part of the epigenome, the inheritance and manifestation of traits associated with imprinted genes is regulated through epigenetic marks. The term “imprintome” was first coined to describe a set of “*cis*-acting imprint regulatory elements” [34]. This term refers to the mechanisms needed for modifying expression including DNA methylation and histone modification, which are two such mechanisms that are well established as being required for the appropriate maintenance of imprinted gene expression [35]. The imprintome is vulnerable to the environment and potentially modified by a host of environmental chemicals and contaminants [36].

Many imprinted genes are grouped in clusters and possess imprinting control regions (ICRs) or a central control region [37]. These ICRs, as well as other regulatory regions associated with imprinted genes, are referred to as differentially methylated regions (DMRs) and display ~50% methylation, where one of the parental alleles is methylated and the other unmethylated in a manner based on parent of origin. These DMRs represent discrete DNA elements that carry a heritable epigenetic mark that distinguishes the parental alleles.

### 1.2. Evolution of Imprinted Genes: A Fight between the Parental Chromosomes

Perhaps the most widely accepted hypothesis for evolutionary underpinnings of the origins of genomic imprinting is the parental conflict theory or the “battle of the sexes” [38,39]. Central to this hypothesis is the struggle to control maternal resources during fetal development, with paternal genes favoring increased use of maternal resources in order to promote the fittest possible offspring, and to divert resources from offspring of other males. Contextually, this hypothesis posits that imprinting arose during early mammalian evolution, where females were able to simultaneously gestate offspring from multiple males. The basis then for the desired growth and resource extraction of the offspring carrying the male’s DNA is an attempt to out-compete offspring from other males. In contrast, maternal genes will suppress fetal growth to ensure equal reproductive success among all her offspring. Supporting this argument, many paternally expressed genes are growth promoting and metabolism-related, whereas maternally expressed genes tend to be growth limiting. The placenta, which can serve to regulate nutrients and growth for the developing fetus, is thought to play a pivotal role in this maternal-paternal fight over resources and control of fetal growth [40]. Within the placenta, a large number of the known imprinted genes are expressed, and genomic imprinting has been confirmed in all placental mammals studied thus far [41].

### 1.3. Imprinted Genes and Their Relationship to Human Health

Imprinted genes have been associated with various human adverse outcomes including diabetes, cancer, developmental disorders, behavioral disorders, and reproductive diseases [42,43]. Prader-Willi and Angelman syndromes were the first disorders to suggest an imprinted mechanism. Though each syndrome manifests differently, it was shown they both arise from deletions in the same region of chromosome fifteen [44]; Prader-Willi results from the loss of a cluster of paternally expressed genes, whereas Angelman syndrome results from the loss of maternal expression within the 15q11-q13 region.

Imprinted genes have also been associated with altered cellular growth resulting in cancer [45,46]. Imprinted genes are particularly vulnerable because they are functionally haploid, thus any epigenetic or genetic perturbations may have a greater impact. For example, epigenetically-induced silencing of the active allele of an imprinted tumor suppressor gene could result in complete loss of expression which in turn would influence cell growth or proliferation [47]. Conversely, epigenetic alterations can also result in activation of the otherwise silent copy of an imprinted growth-promoting gene, contributing to loss of growth regulation. Both of these types of alterations are referred to as “loss of imprinting” (LOI). In fact, LOI has been found across a broad spectrum of tumors and is one of the most common alterations in cancer [46]. As examples, in cancer, the active copy of tumor suppressor cyclin-dependent kinase inhibitor 1C (*CDKN1C*) is frequently aberrantly silenced and the silent copy of the growth promoting insulin-like growth factor II (*IGF2*) gene is often inappropriately activated [48].

### 1.4. Links between Imprinted Genes and Toxic Environmental Metals

Environmental contaminants are currently estimated to be responsible for almost five million deaths and over eighty million Disability-Adjusted Life Years (DALYs) globally [49], and are thought to be involved in 13% to 37% of the global disease burden [50]. Toxic metals represent some of the highest priority contaminants as determined by the Agency for Toxic Substances and Disease Registry (ATSDR) [51].

A number of studies have observed links between exposure to toxic metals and epigenetic events tied to imprinted genes. For example, quantitative analysis was conducted on multiple imprinted gene DMRs in peripheral blood from individuals followed as part of the Cincinnati Lead Study [52]. The researchers identified early childhood lead exposure was associated with hypomethylation of the gene pleiomorphic adenoma gene-like 1 (*PLAGL1*) [53]*.* Prenatal lead exposure has also been linked to decreases in global DNA methylation in cord blood [54]. In addition, lead has been shown to disrupt global DNA methylation patterns in embryonic stem cells [55].

Similarly, exposure to arsenic and cadmium has been observed to alter the methylation of both experimentally validated and predicted imprinted genes. Specifically, in adults exposed to inorganic arsenic, the known imprinted gene anoctamin 1, calcium activated chloride channel (*ANO1*) and predicted imprinted gene forkhead box F1 (*FOXF1*) shows increased promoter methylation in leukocytes [56]. Conversely, in a separate study, the imprinted gene insulin (*INS*) exhibits decreased promoter methylation as a result of arsenic exposure [57]. In a cohort of mother-newborn pairs, the putative imprinted gene zic family member 1 (*ZIC1*), as well as *ANO1*, are differentially methylated in leukocytes, where cadmium exposure was associated with hypermethylation of *ZIC1* in mothers and *ANO1* in newborns [58].

Tobacco is a common source of cadmium exposure [59,60]. Tobacco smoke exposure in utero has been associated with altered DNA methylation [61,62]. These studies showed that among smoking mothers, ten imprinted genes including aryl-hydrocarbon receptor repressor (AHRR), growth factor independent 1 transcription repressor (GFI1), and cytochrome P450, family 1, subfamily A, polypeptide 1 (CYP1A1) were hypomethylated in newborn cord blood [61]. Additionally, the imprinted gene *IGF2* was found to be hypermethylated in cord blood of newborns born to smoking mothers [62]. Infant gender was correlated with differential methylation, as males exhibited smoking related methylation changes at *IGF2* while female newborns did not [62]. While the mechanisms of such sex-associated methylation patterning are unknown, other studies have shown similar sex-differentiated changes in methylation [63,64]. Further research is needed to understand gender specific epigenetic alterations, including imprinting, and may help to inform both sex-linked susceptibility to disease, as well as potentially being predictive of severity and/or prognosis. Additionally, research into the biological basis underlying the roll an individual’s sex plays in disease development may afford the ability to develop targeted therapies based on differential responses.

### 1.5. Study Aim

In the present study, we set out to analyze imprinted genes for their involvement in shared biological pathways and to determine their known interactions with arsenic, cadmium, and lead. This analysis included an assessment of: (i) known relationships between the proteins encoded by the imprinted genes; (ii) common functionality of the proteins encoded by the imprinted genes in the cell; and (iii) common transcription factor-based regulatory regions present in the promoter regions of the imprinted genes.

## 2. Methods

### Imprinted Gene List and Network, Pathway, and Functional Enrichment Analysis

We analyzed imprinted genes derived from a publically available database from GeneImprint [65] that were filtered for experimentally validated and computationally predicted imprinted genes. The genes within the GeneImprint database are classified as predicted based upon chromosomal location [66]. From those, individuals have confirmed some to be imprinted based on actual experiments with cDNA showing parent-of-origin monoallelic expression from humans or other methodologies. Specifically, of the 197 genes, 90 were experimentally confirmed. These two imprinted gene sets were further analyzed for known metals relationships using the comparative toxicogenomics database (CTD) resulting in two additional gene lists with *n* = 43, and *n* = 14, respectively. Toxic metals were prioritized based on their 2011 ATSDR rankings [51]. Metal and imprinted gene relationships were determined using the CTD [67]. The CTD is a public resource that synthesizes current scientific literature on interactions between chemicals, genes, proteins, and the diseases associated with each. However, it should be noted that while the CTD is a useful tool that may be utilized to query a centralized public repository for known gene-metal interactions, the database may be limited in use for emerging findings, as there may be a delay between recent publications and inclusion in the database.

In order to identify biological pathways enriched within the imprinted gene sets, the four gene lists were analyzed for enrichment using Ingenuity Pathway Analysis (IPA) [68]. Ingenuity allows for the mapping of genes, proteins, and their corresponding regulatory networks as a useful tool for the identification of molecular pathways in disease. As a secondary method, for verification, The Database for Annotation, Visualization and Integrated Discovery (DAVIDv6.7) was also used to analyze the four gene lists.

As a method to identify transcription factor binding site enrichment within the imprintome, the imprinted gene set(s) were analyzed using the Genomatix Matinspector module (Genomatix Software Inc., Ann Arbor, MI, USA) [69]. The analysis was used to examine the four gene sets for common regulatory sequences and/or known regulation by common transcription factors. Where multiple promoter regions were possible for a given gene, a single promoter region was selected to maximize the number of experimentally verified 5' complete transcripts. The promoter regions were analyzed with the additional search criteria of 1000 base pairs upstream, and 50 base pairs downstream relative to the transcription start site. The genes were analyzed with a minimum core and matrix similarity of 1.00, the highest level of sensitivity possible. The *p*-value generated is the probability to obtain an equal or greater number of sequences with a match in a randomly drawn sample of the same size as the input sequence set. The lower this probability the higher is the importance of the observed common transcription factor.

## 3. Results and Discussion

### 3.1. Enriched Biological Trends within the Imprinted Gene Set

We analyzed imprinted genes derived from a publically available database that were filtered for experimentally validated and computationally predicted imprinted genes. Of the 197 genes, 90 were experimentally confirmed. These gene lists can be found in Table S1. These two imprinted gene sets were further analyzed for metals relationships using the CTD. Specifically, these associations include cellular perturbations such as altered mRNA and/or protein expression as well as epigenetic modifications (e.g., DNA methylation). The analysis identified known metals interactions for *n* = 43 predicted and experimentally validated imprinted genes and *n* = 14 experimentally validated imprinted genes with known metals interactions (Table S2).

The resulting four imprinted gene lists (both computationally predicted and validated) and the metals-associated genes were analyzed using IPA for known molecular interactions and for enrichment of diseases and canonical pathways. Many of the genes are involved in gene expression, embryonic development, organismal development, gastrointestinal disease, endocrine system disorders, hereditary disorders, cell morphology, cellular development, cell growth and proliferation, cell death and survival (Table 1). The results of IPA analysis were supported by analysis performed using DAVID which found similar enrichment of several of the functional pathways such as embryonic development and gene regulation (Table 1).

In addition, enrichment analyses for canonical pathways were performed using IPA. The imprinted gene set is enriched for two canonical pathways, namely the TP53 and aryl-hydrocarbon receptor (AhR) signalling pathways (Table 1). A total of five genes were associated with TP53 including cyclin-dependent kinase 4 (*CDK4*), *PLAGL1*, retinoblastoma 1 (*RB1*), tumor protein p73 (*TP73*), and Wilms tumor 1 (*WT1*) (enrichment *p* value = 0.00107). A total of five genes were also associated with AhR including aldehyde dehydrogenase 1 family, member L1 (*ALDH1L1*), *CDK4*, cytochrome P450, family 1, subfamily B, polypeptide 1 (*CYP1B1*), *RB1*, and *TP73* (enrichment *p* value < 0.01). The following three genes were common to both pathways: *CDK4*, *RB1*, and *TP73*. This shared gene set perhaps is not surprising, as imprinted genes play a large role in development, and these pathways are fundamental in regulating development, signaling, and cellular responses to stress. However, it should be noted that the observation of the links between the imprinted gene set and these two critical biological pathways has not been previously reported.

Enrichment analyses for canonical pathways were also performed for the experimentally validated imprinted gene set (*n* = 90), where the TP53 signalling pathway was enriched (*p* ≤ 0.001) represented by the following four genes *PLAGL1*, *RB1*, *TP73*, and *WT1*. Imprinted genes involved in the AhR signalling pathway were also present including *RB1* and *TP73*, and the pathway was marginally significant (*p* = 0.09). Further enrichment analysis was performed on the two imprinted gene sets filtered for known association with our prioritized metals; those genes that are predicted or experimentally validated (*n* = 43), and just those experimentally validated (*n* = 14). The TP53 signaling pathway was also enriched in both the predicted/validated and experimentally validated gene sets (*p* < 5.7 × 10^−5^, and *p* < 0.0023, respectively). Enrichment of the AhR signaling pathway was significant (*p* < 0.00023) represented by the genes *CDK4*, *CYP1B1*, *RB1*, and *TP73* in the computationally predicted/experimentally validated gene set, but not in the set of only experimentally validated metal-associated imprinted genes.

**Table 1:** Summary of enriched biological processes/functions of the imprinted gene set.

Networks	*p*-value
Embryonic Development, Organismal Development, Gene Expression *	1 × 10^−39^
Embryonic Organ Development **	1.7 × 10^−12^
Gene Expression, Developmental Disorder, Endocrine System Disorders *	1 × 10^−39^
**Diseases and Disorders**	**Average *p*-value**
Developmental Disorder *	0.001
Endocrine System Disorders *	0.001
Organismal Injury and Abnormalities *	0.002
Gastrointestinal Disease *	0.002
Hereditary Disorder *	0.006
Prader-Willi Syndrome **	0.02
Beckwith-Wiedemann Syndrome **	0.0007
**Molecular and Cellular Functions**	**Average *p*-value**
Gene Expression *	^<0.001^
Transcription Regulation **	8.2 × 10^−8^
Cell Morphology *	0.002
Cell Morphogenesis Involved in Differentiation	0.005
Cellular Development *	0.003
Development-associated Proteins **	1.8 × 10^−10^
Cell Death and Survival *	0.003
Cell Signaling *	0.003
**Canonical Pathways**	***p*-value**
TP53 Signaling *	0.001
Aryl Hydrocarbon Receptor Signaling *	0.006

(*) Ingenuity Pathway Analysis (IPA) results; (**) Database for Annotation, Visualization and Integrated Discovery (DAVID) results.

#### 3.1.1. TP53 Signaling-Associated Imprinted Genes

The TP53 tumor suppressor protein is known as the guardian of the genome. It is a key transcriptional regulator that responds to a variety of cellular stresses including damage induced by various environmental contaminants. It serves to control key cellular processes such as DNA repair, cell-cycle progression, angiogenesis, and apoptosis pathways critical for influencing apoptosis or cell-cycle arrest [70]. In addition to its critical roles in DNA repair pathways, TP53 can act as a transcriptional regulator. Mutations within the *TP53* gene are responsible for Li-Fraumeni syndrome [71] and loss in functionality of the tumor suppressor is thought to be a contributing factor in the majority of cancer cases [72,73].

Of the five imprinted genes found to be associated with TP53, TP73 has been shown to increase activation of phosphorylated TP53 in mouse embryo fibroblasts [74] and PLAGL1 was shown to act as a transcriptional co-activator and enhance the activity of TP53 in both human carcinoma P53^+/−^ and HeLa cells [75]. In *in vitro* models of rat kidney and human osteoblast-like cells, binding of WT1 to TP53 has been shown to stabilize TP53, as well as inhibit TP53-mediated apoptosis [76]. Further, TP53 has been shown to modulate CDK4/RB1 through CDKN1A in human colon cancer cells [77]. TP53 can increase the expression of CDKN1A, which in turn decreases phosphorylation of RB1 [78]. Additionally, CDKN1A may also decrease phosphorylation of RB1 via CDK2-Cyclin D1 complex [79].

#### 3.1.2. AhR Signaling-Associated Imprinted Genes

The aryl-hydrocarbon receptor is a transcription factor involved in cell cycle regulation, and an initiator of biological responses to xenobiotics. AhR has been shown to regulate enzymes such as cytochrome P450 and other xenobiotic metabolizing enzyme genes including putative imprinted gene *CYP1B1* [80]. In addition to xenobiotic metabolism, the AhR pathway plays a key role in organismal development processes [81]. In vertebrates, AhR is important for cellular proliferation and differentiation as well as many developmental pathways [82]. In addition to its role in mediating xenobiotics, the AhR pathway contributes to gene regulation and carcinogenesis [83].

Of the five genes found to be associated with AhR, CDK4 is involved in regulation of RB1 through phosphorylation [84]. It has been shown that RB1 increases activation of the AhR-Arnt complex in hepatoma cells [85] and this heterodimer complex is known to regulate CYP1B1 [86] and ALDH1 [87]. AhR has also been shown to decrease activation of phosphorylated E2F1 in tandem with RB1 [88,89]. This is worth noting, as expression of *TP73*, as well as many cyclins, is regulated by E2F1 [90,91].

#### 3.1.3. Relationships between Imprinted Genes and Toxic Metals

**Figure 1:**
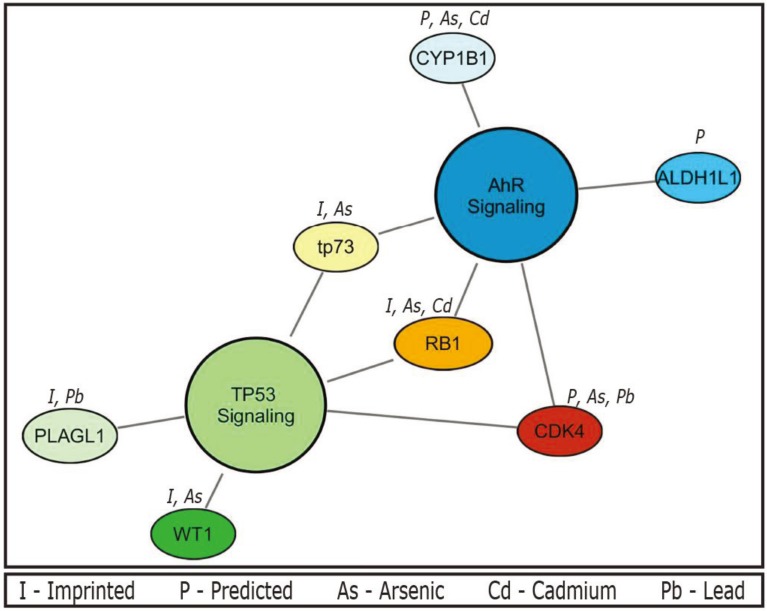
Top canonical pathways and their relationships to toxic metals. The aryl-hydrocarbon receptor (AhR) (*p* = 0.001) and TP53 (*p* = 0.007) networks display known interactions between pathway genes and priority metals (arsenic, cadmium and lead). Imprinted status is also noted; abbreviations are shown in the figure legend.

Using data collected from the CTD, the seven unique genes within the TP53 and AhR pathways were detailed according to their known relationships to metals (Figure 1). The current body of literature pertaining to these relationships includes associations of the metals with altered gene regulation. Of the three genes (*CDK4*, *RB1*, and *TP73*) common to both pathways, upregulation of *CDK4* is observed upon exposure to both arsenic in a glioma cell line [92] and lead measured in peripheral blood [93]. Similarly, exposure to inorganic arsenic is associated with increased TP73 protein expression and activation of the downstream TP73-TP53 pathway in leukemia cells [94]. Conversely, there is decreased expression of *RB1* from arsenic [95] and cadmium exposure [96].

Of the genes specific to the TP53 pathway, exposure to inorganic arsenic is associated with decreased expression of the *WT1* gene in leukemia cell lines [97]. To date, the only known interaction between *PLAGL1* and environmental metals is hypomethylation associated with lead exposure [53]. Importantly, changes to signaling within the TP53 pathway by environmental contaminants such as inorganic arsenic and cadmium can impact the balance between apoptosis and proliferation in epithelial cancer cell lines [98,99].

Gene-metal interactions that disrupt the AhR pathway can disturb xenobiotic metabolism and increase an individual’s susceptibility to a range of negative health outcomes. Studies have shown decreased expression of *CYP1B1* in response to metals such as arsenic [100] and cadmium in acute promyelocytic leukemia cell lines [101]. Interactions between *ALDH1L1* and environmental metals have not been studied. It should be mentioned that the analyses for gene-metal interactions were performed using the CTD database, which may not be fully comprehensive due to querying limitations and time delays between published findings and inclusion in the database. Tools such as CTD or IPA’s canonical pathway analysis may be prone to inherent selection bias. Resources such as these that index interactions from published research may disproportionately display enrichment for a particular outcome or pathway as a consequence of overrepresentation of that particular interaction within the literature. Nevertheless, the alteration of these putative imprinted genes within the AhR pathway by inorganic arsenic, cadmium, and possibly other toxic metals may then serve as a potential mechanism of later life health outcomes including cancers and susceptibility to exogenous chemicals.

### 3.2. Sequence Specific Patterns of TF Elements in the Imprinted Genes

An analytical method was used to explore the four separate gene sets including experimentally validated and predicted imprinted genes (*n* = 197), experimentally validated imprinted genes (*n* = 90), experimentally validated and predicted imprinted genes associated with metals enrichment (*n* = 43), and experimentally validated imprinted genes associated with metals enrichment (*n* = 14) for common regulatory sequences and/or known regulation by common transcription factors. These results demonstrated that two transcription factor families were identified across all four gene lists, namely the family containing TF2B (Transcription Factor II B) and the PLAG family (Table 2).

**Table 2:** Enriched transcription factors amongst the imprinted gene sets.

Gene set	Transcription factor families	Transcription factors	Genes	*p*-value	Representative consensus sequence
**Experimentally Validated & Predicted IGs (*n* = 197)**	TF2B	GTF2B	93/171 (54%)	3.79E-45	ccgCGCC ^1^
	PLAG	PLAG1 PLAGL1 PLAGL2	133/171 (78%)	2.10E-08	gaGGGGgcggggggggggggggg ^2^
**Experimentally Validated IGs (*n* = 90)**	TF2B	GTF2B	38/71 (54%)	4.72E-19	ccgCGCC
	PLAG	PLAG1 PLAGL1 PLAGL2	55/71 (77%)	3.48E-04	gaGGGGgcggggggggggggggg
**Experimentally Validated & Predicted IGs Metals-associated (*n* = 43)**	TF2B	GTF2B	21/43 (49%)	1.09E-10	ccgCGCC
	PLAG	PLAG1 PLAGL1 PLAGL2	35/43 (81%)	1.31E-04	gaGGGGgcggggggggggggggg
**Experimentally Validated IGs Metals-associated (*n* = 14)**	TF2B	GTF2B	7/14 (50%)	2.62E-04	ccgCGCC
	PLAG	PLAG1 PLAGL1 PLAGL2	13/14 (93%)	4.90E-03	gaGGGGgcggggggggggggggg

^1^ Consensus sequence based on most conserved nucleotide at each position for 210 sequences; ^2^ Consensus sequence based on most conserved nucleotide at each position for 337 sequences.

TF2B is a transcription factor that mediates interactions between RNA polymerase II and promoter regions [102]. The other significantly enriched transcription factor family was the PLAG family. The PLAG family contains the transcription factors pleiomorphic adenoma gene 1 (PLAG1), pleiomorphic adenoma gene-like 2 (PLAGL2), and Pleiomorphic Adenoma Gene-Like 1 (PLAGL1, also known as ZAC1) encoded by the imprinted gene *PLAGL1*.

Interestingly, PLAGL1 is a zinc finger protein transcription factor that has been implicated as a regulatory hub in an “imprinted gene network” (IGN) controlling embryonic growth and cell proliferation, and proposed as a regulator of expression of other imprinted genes including *IGF2*, *H19*, and *CDKN1C* [103,104]. The data here expand upon this notion and in fact support that many, specifically 133 of the 171 analyzed imprinted genes (*i.e*., 77%–78%) have binding sites for PLAG transcription factors, thus greatly expanding the current list of potential imprinted gene targets of PLAG.

Likewise, when only the metal-associated imprinted genes (*n* = 14) were analyzed, the most significantly enriched transcription factor families were once again the families of *TF2B* (*p* < 2.62 × 10^−4^) and *PLAG* (*p* < 4.9 × 10^−3^). TF2B motifs were enriched in 7 of 14 (50%) metal associated gene sequences.

Transcription factors are known regulators of gene expression but they also serve an important role in regulating access to the DNA within genes. This access has consequences for both transcription and DNA methylation patterning. The binding of transcription factors to specific sites within the promoter region may protect CpG islands from methylation [105]. Recent work has found that areas with high transcription factor binding tend to have lower methylation [106].

Expanding upon previous work linking transcription factor occupancy with footprints left by DNase 1 [107,108], the Stamatoyannopoulos group studied numerous cell and tissue lines and found such footprint occupancy represented a viable quantitative measure of transcription factor occupancy and such occupancy afforded protection from DNA methylation [109].

In this context, our laboratory has recently hypothesized that in the case of cadmium and likely other environmental contaminants as well, distinct methylation patterns may represent “environmental footprints” or indicators of transcription factor occupancy during times of DNA methylation [58]. Based on the results from the present study, it can be hypothesized that specific transcription factor families may impact occupancy related to imprinted gene regulatory regions and subsequently their potential for DNA methylation. The identification of these regulators of the methylation “footprints” may serve as important biomarkers of environmental exposure, and may help to support a mechanistic link between toxic metals exposure, imprinted gene alterations, and later life health outcomes.

## 4. Conclusions and Future Research Directions

Using a survey of current experimentally validated and predicted imprinted genes, we set out to examine shared functionality and pathway enrichment within the imprinted gene set. Notably, we found: (i) common functionality among proteins encoded by the imprinted gene set; (ii) several of the putative and known imprinted genes play a role in the TP53 and AhR pathways and many of these imprinted genes have been shown to interact with toxic environmental metals specifically in their ability to modify, and in certain cases disrupt, apoptosis, cell cycle arrest, ligand metabolism, DNA repair, and may promote the development of certain cancers; and (iii) common transcription factor-based regulatory regions for TF2B and PLAG present in the promoter regions of the experimentally validated and predicted imprinted genes.

As a result of their impact of access to DNA, transcription factors may contribute to specific DNA methylation patterning upon exposure to metals. Our lab has recently hypothesized that distinct methylation patterns due to exposure to environmental contaminants may represent “environmental footprints” of transcription factor occupancy during DNA methylating events. Based on our results, it can be hypothesized that specific transcription factor families such as TF2B and PLAG may impact the occupancy and subsequent methylation of the imprinted gene set. Additional research is needed to understand the mechanisms linking metals exposure, transcription factor occupancy and the imprintome. It is also worth noting that we prioritized the current research on cadmium, lead, and arsenic. While these metals represent some of the most studied, the lack of inclusion the emerging roll additional metals play in terms of impact on the epigenome is not a reflection of their toxicity, or relationship to genetic imprinting. Furthermore, metals are only one class of the broad range of environmental contaminants that have been shown, or may be associated with genetic imprinting and other epigenetic alterations.

Our novel finding that *CDK4*, *RB1*, and *TP73* are associated with two biologically critical pathways, TP53 and AhR may have implications for understanding the biological mechanisms of how these imprinted genes may ultimately be associated with negative health outcomes. Importantly, as more databases are populated with published work on imprinted genes and their interactions with toxic metals, other statistically significant pathways may be associated with the imprintome, or subsets of imprinted genes, and may provide further mechanistic links between genetic imprinting and disease.

Furthermore, our current findings have implications for understanding perturbed biological pathways that may provide insight into early life exposures and later life health consequences. In addition, it is worth noting, that while the idea is still under debate, further studies are needed to determine the extent of trans-generational inheritability of specific patterns of methylation and their contributions to disease. Combined with the dysregulation of imprinted genes by toxic-metals, it may be possible to link ancestral exposure to environmental contaminants with current patterns of disease.

Based on our findings, further research is recommended to investigate the biological consequences of the imprinted gene set and its relationship to transcription factor occupancy. Specifically, transcription factor knockdown experiments or controlled toxicological experiments may help to elucidate the relationship between imprinted genes, transcription factor occupancy, environmental exposures, and associated health consequences.

## Supplementary Files

Supplementary File 1 Supplementary Information (PDF, 315 KB)
