# Supplementary material for: Imprinted Genes and the Environment: Links to the Toxic Metals Arsenic, Cadmium and Lead
Source: Genes (Basel). 2014 Jun 11;5(2):477–96. doi: 10.3390/genes5020477 (PMC4094944; doi:10.3390/genes5020477)
Supplement: Supplementary File 1 — Supplementary Information (PDF, 315 KB) [file genes-05-00477-s001.pdf]

Supplementary Information

Table S1. Confirmed and predicted Imprinted Genes.

Gene ID	Full Gene Name	Location	Status	Expressed Allele
ABCC9	ATP-binding cassette, sub-family C, member 9	12p12.1 AS	Predicted	Maternal
ABCG8	ATP-binding cassette, sub-family G, member 8	2p21	Predicted	Maternal
ACD	Adrenocortical dysplasia homolog	16q22.1 AS	Predicted	Maternal
ADAMTS16	ADAM metallopeptidase with thrombospondin type 1 motif, 16	5p15	Predicted	Maternal
AIM1	Absent in melanoma 1	6q21	Imprinted	Paternal
ALDH1L1	Aldehyde dehydrogenase 1 family, member L1	3q21.3 AS	Predicted	Maternal
ANO1	Anoctamin 1, calcium activated chloride channel	11q13.3	Imprinted	Maternal
APBA1	Amyloid beta precursor protein-binding, family A, member 1	9q13-q21.1 AS	Predicted	Paternal
ATP10A	ATPase, class V, type 10A	15q11.2 AS	Imprinted	Maternal
B4GALNT4	Beta-1,4-N-acetyl-galactosaminyl transferase 4	11p15.5	Predicted	Maternal
BLCAP	Bladder cancer associated protein	20q11.2-q12 AS	Imprinted	Isoform Dependent
BMP8B	Bone morphogenetic protein 8b	1p35-p32 AS	Predicted	Paternal
BRP44L	Brain protein 44-like	6q27 AS	Predicted	Paternal
BRUNOL4	Bruno-like 4, RNA binding protein	18q12 AS	Predicted	Maternal
BTNL2	Butyrophilin-like 2	6p21.3 AS	Predicted	Maternal
C10orf91	Chromosome 10 open reading frame 91	10q26.3	Predicted	Maternal
C10orf93	Chromosome 10 open reading frame 93	10q26.3 AS	Predicted	Maternal
C16orf57	Chromosome 16 open reading frame 57	16q21	Predicted	Maternal
C20orf20	Chromosome 20 open reading frame 20	20q13.33	Predicted	Maternal
C20orf82	Chromosome 20 open reading frame 82	20p12.1	Predicted	Paternal
C6orf117	Chromosome 6 open reading frame 117	6q14.3	Predicted	Paternal
C9orf116	Chromosome 9 open reading frame 116	9q34.3 AS	Predicted	Paternal
C9orf85	Chromosome 9 open reading frame 85	9q21.13	Predicted	Paternal
CCDC85A	Coiled-coil domain containing 85A	2p16.1	Predicted	Paternal
CDH18	Cadherin 18, type 2	5p15.2-p15.1 AS	Predicted	Paternal
CDK4	Cyclin-dependent kinase 4	12q14 AS	Predicted	Maternal
CDKN1C	Cyclin-dependent kinase inhibitor 1C	11p15.5 AS	Imprinted	Maternal
CHMP2A	Chromatin modifying protein 2A	19q AS	Predicted	Maternal
CHST8	Carbohydrate sulfotransferase 8	19q13.1	Predicted	Maternal
COL9A3	Collagen, type IX, alpha 3	20q13.3	Predicted	Maternal
COPG2IT1	COPG2 imprinted transcript 1	7q32	Imprinted	Paternal
CPA4	Carboxypeptidase A4	7q32	Imprinted	Maternal
CSF2	Colony stimulating factor 2	5q31.1	Predicted	Maternal
CYP1B1	Cytochrome P450, family 1, subfamily B, polypeptide 1	2p21 AS	Predicted	Paternal
DDC	Dopa decarboxylase	7p12.2 AS	Imprinted	Isoform Dependent
DGCR6	DiGeorge syndrome critical region gene 6	22q11.21	Imprinted	Unknown
DGCR6L	DiGeorge syndrome critical region gene 6-like	22q11 AS	Imprinted	Unknown
DIRAS3	DIRAS family, GTP-binding RAS-like 3	1p31 AS	Imprinted	Paternal
DLGAP2	Discs, large homolog-associated protein 2	8p23	Imprinted	Paternal

Table S1. *Cont.*

Gene ID	Full Gene Name	Location	Status	Expressed Allele
DLK1	Delta-like 1 homolog	14q32	Imprinted	Paternal
DLX5	Distal-less homeobox 5	7q22 AS	Imprinted	Maternal
DNMT1	DNA (cytosine-5-)-methyltransferase 1	19p13.2 AS	Imprinted	Paternal
DVL1	Dishevelled, dsh homolog 1	1p36 AS	Predicted	Maternal
E2F7	E2F transcription factor 7	12q21.2 AS	Predicted	Maternal
EGFL7	EGF-like-domain, multiple 7	9q34.3	Predicted	Paternal
EVX1	Even-skipped homeobox 1	7p15-p14	Predicted	Paternal
FAM50B	Family with sequence similarity 50, member B	6p25.2	Imprinted	Paternal
FAM59A	Family with sequence similarity 59, member A	18q12.1 AS	Predicted	Paternal
FAM70B	Family with sequence similarity 70, member B	13q34	Predicted	Maternal
FASTK	Fas-activated serine/threonine kinase	7q35 AS	Predicted	Maternal
FBRSL1	Fibrosin-like 1	12q24.33	Predicted	Maternal
FERMT2	Fermitin family homolog 2	14q22.1 AS	Predicted	Paternal
FGFRL1	Fibroblast growth factor receptor-like 1	4p16	Predicted	Maternal
FLJ20464	Hypothetical protein FLJ20464	22q12.2	Predicted	Paternal
FLJ40296	FLJ40296 protein	13q21.1	Predicted	Maternal
FLJ46321	FAM75-like protein FLJ46321	9q21.32	Predicted	Maternal
FOXF1	Forkhead box F1	16q24	Predicted	Maternal
FOXG1	Forkhead box G1	14q13	Predicted	Paternal
FUCA1	Fucosidase, alpha-L-1, tissue	1p34 AS	Predicted	Paternal
GATA3	GATA binding protein 3	10p15	Predicted	Paternal
GFI1	Growth factor independent 1 transcription repressor	1p22 AS	Predicted	Paternal
GLI3	GLI family zinc finger 3	7p13 AS	Predicted	Maternal
GLIS3	GLIS family zinc finger 3	9p24.2 AS	Imprinted	Paternal
GNAS	GNAS complex locus	20q13.3	Imprinted	Isoform Dependent
GNASAS	GNAS antisense RNA	20q13.32 AS	Imprinted	Paternal
GPR1	G protein-coupled receptor 1	2q33.3 AS	Imprinted	Paternal
GPT	Glutamic-pyruvate transaminase	8q24.3	Predicted	Maternal
GRB10	Growth factor receptor-bound protein 10	7p12-p11.2 AS	Imprinted	Isoform Dependent
H19	H19, imprinted maternally expressed untranslated mRNA	11p15.5 AS	Imprinted	Maternal
HES1	Hairy and enhancer of split 1,	3q28-q29	Predicted	Paternal
HIST3H2BB	Histone cluster 3, H2bb	1q42.13	Predicted	Maternal
HOXA11	Homeobox A11	7p15-p14 AS	Predicted	Maternal
HOXA2	Homeobox A2	7p15-p14 AS	Predicted	Maternal
HOXA3	Homeobox A3	7p15-p14 AS	Predicted	Maternal
HOXA4	Homeobox A4	7p15-p14 AS	Predicted	Maternal
HOXA5	Homeobox A5	7p15-p14 AS	Predicted	Maternal
HOXB2	Homeobox B2	17q21-q22 AS	Predicted	Maternal
HOXB3	Homeobox B3	17q21.3 AS	Predicted	Maternal
HOXC4	Homeobox C4	12q13.3	Predicted	Maternal
HOXC9	Homeobox C9	12q13.3	Predicted	Maternal

Table S1. *Cont.*

Gene ID	Full Gene Name	Location	Status	Expressed Allele
HSPA6	Heat shock 70 kDa protein 6	1q23	Predicted	Maternal
HYMAI	Hydatidiform mole associated and imprinted	6q24.2 AS	Imprinted	Paternal
IFITM1	Interferon induced transmembrane protein 1	11p15.5	Predicted	Maternal
IGF2	Insulin-like growth factor 2	11p15.5 AS	Imprinted	Paternal
IGF2AS	Insulin-like growth factor 2 antisense	11p15.5	Imprinted	Paternal
INPP5F	inositol polyphosphate-5-phosphatase F	10q26.11	Imprinted	Paternal
INS	Insulin	11p15.5 AS	Imprinted	Paternal
ISM1	Isthmin 1 homolog	20p12.1	Predicted	Paternal
KBTBD3	Kelch repeat and BTB domain containing 3	11q22.3 AS	Predicted	Paternal
KCNK9	Potassium channel, subfamily K, member 9	8q24.3 AS	Imprinted	Maternal
KCNQ1	Potassium voltage-gated channel, KQT-like subfamily, member 1	11p15.5	Imprinted	Maternal
KCNQ1DN	KCNQ1 downstream neighbor	11p15.4	Imprinted	Maternal
KCNQ1OT1	KCNQ1 overlapping transcript 1	11p15	Imprinted	Paternal
KIAA1530	UV-Stimulated Scaffold Protein A	4p16.3	Predicted	Maternal
KIAA1545	KIAA1545 protein	12q24.33	Predicted	Maternal
KLF14	Kruppel-like factor 14	7q32.3 AS	Imprinted	Maternal
L3MBTL	L(3)mbt-like	20q13.12	Imprinted	Paternal
LDB1	LIM domain binding 1	10q24-q25 AS	Predicted	Maternal
LILRB4	Leukocyte immunoglobulin-like receptor, subfamily B, member 4	19q13.4	Predicted	Maternal
LIN28B	Lin-28 homolog B	6q21	Imprinted	Paternal
LMX1B	LIM homeobox transcription factor 1, beta	9q34	Predicted	Maternal
LOC100131170	Hypothetical LOC100131170	17q25.3 AS	Predicted	Paternal
LRRTM1	Leucine rich repeat transmembrane neuronal 1	2p12 AS	Imprinted	Paternal
LY6D	Lymphocyte antigen 6 complex, locus D	8q24-qter AS	Predicted	Paternal
MAGEL2	MAGE-like 2	15q11-q12 AS	Imprinted	Paternal
MAGI2	Membrane associated guanylate kinase, WW and PDZ domain containing 2	7q21 AS	Imprinted	Maternal
MCTS2	20q11.21	20q11.21	Imprinted	Paternal
MEG3	Maternally expressed 3	14q32	Imprinted	Maternal
MEST	Mesoderm specific transcript homolog	7q32	Imprinted	Paternal
MESTIT1	MEST intronic transcript 1, antisense RNA	7q32.2 AS	Imprinted	Paternal
MIMT1	MER1 repeat containing imprinted transcript 1	19q13.4	Imprinted	Paternal
MIR296	MicroRNA 296	20q13.32 AS	Imprinted	Paternal
MIR298	MicroRNA 298	20q13.32 AS	Imprinted	Paternal
MIR371A	MicroRNA 371a	19q13.42	Imprinted	Paternal
MKRN3	Makorin ring finger protein 3	15q11-q13	Imprinted	Paternal
MRAP2	Melanocortin 2 receptor accessory protein 2	6q14.2	Predicted	Paternal
MYEOV2	Myeloma overexpressed 2	2q37.3 AS	Predicted	Paternal
MZF1	Myeloid zinc finger 1	19q13.4 AS	Predicted	Maternal
NAA60	N(alpha)-acetyltransferase 60, NatF catalytic subunit	16p13.3	Imprinted	Maternal
NAP1L5	Nucleosome assembly protein 1-like 5	4q22.1 AS	Imprinted	Paternal

Table S1. *Cont.*

Gene ID	Full Gene Name	Location	Status	Expressed Allele
NDN	Necdin homolog	15q11.2-q12 AS	Imprinted	Paternal
NDUFA4P1	NADH dehydrogenase 1 alpha subcomplex, 4, 9 kDa, pseudogene 1	1p13.3	Predicted	Paternal
NKAIN3	Na+/K+ transporting ATPase interacting 3	8q12.3	Predicted	Paternal
NKX6-2	NK6 homeobox 2	10q26 AS	Predicted	Maternal
NLRP2	NLR family, pyrin domain containing 2	19q13.42	Imprinted	Maternal
NNAT	Neuronatin	20q11.2-q12	Imprinted	Paternal
NPAP1	Nuclear pore associated protein 1	15q11-q13	Imprinted	Unknown
NTM	Neurotrimin	11q25	Imprinted	Maternal
OBSCN	Obscurin, cytoskeletal calmodulin and titin-interacting RhoGEF	1q42.13	Predicted	Paternal
OR11L1	Olfactory receptor, family 11, subfamily L, member 1	1q44 AS	Predicted	Paternal
OSBPL5	Oxysterol binding protein-like 5	11p15.4 AS	Imprinted	Maternal
OTX1	Orthodenticle homeobox 1	2p13	Predicted	Maternal
PAOX	Polyamine oxidase	10q26.3	Predicted	Maternal
PEG10	Paternally expressed 10	7q21	Imprinted	Paternal
PEG3	Paternally expressed gene 3	19q13.4 AS	Imprinted	Paternal
PEX10	Peroxisomal biogenesis factor 10	1p36.32 AS	Predicted	Maternal
PHLDA2	Pleckstrin homology-like domain, family A, member 2	11p15.5 AS	Imprinted	Maternal
PHPT1	Phosphohistidine phosphatase 1	9q34.3	Predicted	Maternal
PKP3	Plakophilin 3	11p15	Predicted	Maternal
PLAGL1	Pleiomorphic adenoma gene-like 1	6q24-q25 AS	Imprinted	Paternal
PPAP2C	Phosphatidic acid phosphatase type 2C	19p13 AS	Predicted	Maternal
PPP1R9A	Protein phosphatase 1, regulatory subunit 9A	7q21.3	Imprinted	Maternal
PRDM16	PR domain containing 16	1p36.23-p33	Predicted	Paternal
PSIMCT-1	Malignant T cell amplified sequence 1 pseudogene	20q11.2	Imprinted	Paternal
PTPN14	Protein tyrosine phosphatase, non-receptor type 14	1q32.2 AS	Predicted	Maternal
PURG	Purine-rich element binding protein G	8p11 AS	Predicted	Paternal
PWCR1	Prader-Willi syndrome chromosome region 1	15q11.2	Imprinted	Paternal
PYY2	Peptide YY, 2	17q11	Predicted	Paternal
RAB1B	RAB1B, member RAS oncogene family	11q12	Predicted	Maternal
RB1	Retinoblastoma 1	13q14.2	Imprinted	Maternal
RBP5	Retinol binding protein 5, cellular	12p13.31 AS	Imprinted	Maternal
RNU5D-1	RNA, U5D small nuclear 1	1p34.1 AS	Imprinted	Paternal
RPL22	Ribosomal protein L22	1p36.3-p36.2 AS	Predicted	Paternal
SALL1	Sal-like 1	16q12.1 AS	Predicted	Maternal
SANG	GNAS1 antisense	20q13.32	Imprinted	Paternal
SGCE	Sarcoglycan, epsilon	7q21-q22 AS	Imprinted	Paternal
SIM2	Single-minded homolog 2	21q22.2	Predicted	Paternal
SLC22A18	Solute carrier family 22, member 18	11p15.5	Imprinted	Maternal
*SLC22A2**	Solute carrier family 22, member 2	6q26 AS	Imprinted	Maternal
*SLC22A3**	Solute carrier family 22, member 3	6q26-q27	Imprinted	Maternal
SLC26A10	Solute carrier family 26, member 10	12q13	Predicted	Maternal

Table S1. *Cont.*

Gene ID	Full Gene Name	Location	Status	Expressed Allele
SLC4A2	Solute carrier family 4, anion exchanger, member 2	7q35-q36	Predicted	Maternal
SNORD107	Small nucleolar RNA, C/D box 107	15q11.2	Imprinted	Paternal
SNORD108	Small nucleolar RNA, C/D box 108	15q11.2	Imprinted	Paternal
SNORD109A	Small nucleolar RNA, C/D box 109A	15q11.2	Imprinted	Paternal
SNORD109B	Small nucleolar RNA, C/D box 109B	15q11.2	Imprinted	Paternal
SNORD115@	Small nucleolar RNA, C/D box 115 cluster	15q11.2	Imprinted	Paternal
SNORD115-48	Small nucleolar RNA, C/D box 115-48	15q11.2	Imprinted	Paternal
SNORD116@	Small nucleolar RNA, C/D box 116 cluster	15q11.2	Imprinted	Paternal
SNORD64	Small nucleolar RNA, C/D box 64	15q12	Imprinted	Paternal
SNRPN	Small nuclear ribonucleoprotein polypeptide N	15q11.2	Imprinted	Paternal
SNURF	SNRPN upstream reading frame	15q12	Imprinted	Paternal
SOX8	SRY-box 8	16p13.3	Predicted	Paternal
SPON2	Spondin 2, extracellular matrix protein	4p16.3 AS	Predicted	Paternal
TCEB3C	Transcription elongation factor B polypeptide 3C	18q21.1 AS	Imprinted	Maternal
TFPI2	Tissue factor pathway inhibitor 2	7q22 AS	Imprinted	Maternal
TIGD1	Tigger transposable element derived 1	2q37.1 AS	Predicted	Paternal
TMEM52	Transmembrane protein 52	1p36.33 AS	Predicted	Paternal
TMEM60	Transmembrane protein 60	7q11.23 AS	Predicted	Paternal
TMEM88	Transmembrane protein 88	17p13.1	Predicted	Maternal
TP73	Tumor protein p73	1p36.3	Imprinted	Maternal
TSHZ3	Teashirt zinc finger homeobox 3	19q12 AS	Predicted	Paternal
UBE3A	Ubiquitin protein ligase E3A	15q11-q13 AS	Imprinted	Maternal
VAX2	Ventral anterior homeobox 2	2p13	Predicted	Maternal
VENTX	VENT homeobox homolog	10q26.3	Predicted	Maternal
WDR8	WD repeat domain 8	1p36.3 AS	Predicted	Maternal
WT1	Wilms tumor 1	11p13 AS	Imprinted	Paternal
ZC3H12C	Zinc finger CCCH-type containing 12C	11q22.3	Imprinted	Paternal
ZDBF2	Zinc finger, DBF-type containing 2	2q33.3	Imprinted	Paternal
ZFAT	Zinc finger and AT hook domain containing	8q24.22 AS	Imprinted	Paternal
ZFAT-AS1	Zinc finger and AT hook domain containing antisense RNA 1	8q24.22	Imprinted	Paternal
ZFP36L2	Zinc finger protein 36, C3H type-like 2	2p22.3-p21 AS	Predicted	Maternal
ZIC1	Zic family member 1	3q24	Predicted	Maternal
ZIM2	Zinc finger, imprinted 2	19q13.4 AS	Imprinted	Paternal
ZNF225	Zinc finger protein 225	19q13.2	Predicted	Paternal
ZNF229	Zinc finger protein 229	19q13.31 AS	Predicted	Maternal
ZNF597	Zinc finger protein 597	16p13.3 AS	Imprinted	Maternal

Table S2. Associations between the imprinted gene set and Arsenic, Cadmium, Lead, and Mercury in human studies curated through the Comparative Toxicogenomics Database.

Gene ID	Metal	Interaction	Status	PMID
ACD	Lead	decreased expression of ACD mRNA	Predicted	19921347
ANO1	Arsenic	increased methylation of ANO1 promoter region	Imprinted	21291286
APBA1	Cadmium	decreased expression of APBA1 mRNA	Predicted	12634122
	Lead	decreased expression of APBA1 mRNA	Predicted	12634122
C9ORF116	Cadmium	increased expression of C9ORF116 mRNA	Predicted	21457566
CDK4	Arsenic	increased expression of CDK4 mRNA	Predicted	20654705<br>17530484<br>19769630<br>20016248
	Lead	increased expression of CDK4 mRNA	Predicted	19921347
CDKN1C	Arsenic	increased expression of CDKN1C mRNA	Imprinted	18585445
CSF2	Arsenic	decreased expression of RELA, CASP3, and CASP8 proteins	Predicted	16174796
		increased expression of CSF2 protein	Predicted	11312651
	Cadmium	increased expression and secretion of CSF2 protein	Predicted	18082304
CYP1B1	Arsenic	increased expression of CYP1B1 mRNA	Predicted	17530438<br>15894607
	Cadmium	decreased expression of CYP1B1 mRNA	Predicted	12760830<br>18560533
DLK1	Arsenic	DLK1 protein affects the susceptibility to Arsenic	Imprinted	18575777
DNMT1	Arsenic	decreased expression of DNMT1 mRNA	Imprinted	23159075<br>20596618<br>21176356<br>16613325
		decreased expression of DNMT1 protein	Imprinted	22558281<br>21687957
	Cadmium	decreased expression of DNMT1 mRNA	Imprinted	22112500
E2F7	Arsenic	decreased expression of E2F7 mRNA	Predicted	19945496
EGFL7	Lead	decreased expression of EGFL7 mRNA	Predicted	19921347
FOXF1	Arsenic	increased methylation of FOXF1 promoter	Predicted	21291286
FUCA1	Arsenic	increased expression of FUCA1 mRNA	Predicted	20458559
GATA3	Arsenic	inhibits the binding of GATA3 and IL13 promoter and KPNA2 protein	Predicted	19080345
GNAS	Lead	increased expression of GNAS mRNA	Imprinted	19921347
GPT	Cadmium	decreased expression of GPT protein	Predicted	11281253
GRB10	Arsenic	increased expression of GRB10 mRNA	Imprinted	20458559
	Arsenic	GRB10 mRNA affects the susceptibility to arsenic	Predicted	23911876
	Cadmium	decreased expression of GRB10 mRNA	Predicted	12160620
HES1	Arsenic	decreases expression of HES1 mRNA	Predicted	23832679<br>20458559<br>23497375<br>18633435
HOXA5	Arsenic	decreased expression of HOXA5 mRNA	Predicted	21461292
HSPA6	Arsenic	increased expression of HSPA6 mRNA	Predicted	19417148<br>16132727<br>15978632

Table S2. *Cont.*

Gene ID	Metal	Interaction	Status	PMID
		increased expression of HSPA6 protein	Predicted	15978632<br>19364129
	Cadmium	Increased expression of HSPA6 mRNA	Predicted	21172416<br>22562489<br>12064557
	Mercury	Increased expression of HSPA6 mRNA	Predicted	22129749
IFITM1	Arsenic	decreased expression of IFITM1 mRNA	Predicted	12890387
IGF2	Cadmium	promotes binding of IGF2 protein to IGF1R protein	Validated	9648927
INS	Arsenic	decreased methylation of INS promoter	Imprinted	23315758
	Cadmium	decreased expression of INS protein	Predicted	17303580
LILRB4	Lead	Altered expression of LILRB4 in relation to cord blood level concentrations	Predicted	22313790
MRAP2	Mercury	MRAP2 protein increases susceptibility to mercury	Predicted	21139347
NDUFA4	Arsenic	increased expression of NDUFA4 mRNA	Predicted	20458559
OSBPL5	Arsenic	altered expression of OSBPL5 mRNA	Imprinted	18414638
PAOX	Arsenic	increased expression of PAOX mRNA	Predicted	15761015
PHLDA2	Arsenic	decreased expression of PHLDA2 mRNA	Imprinted	15761015
PKP3	Arsenic	PKP3 protein results in decreased susceptibility to arsenic	Predicted	20707922
PPAP2C	Arsenic	PPAP2C protein results in decreased susceptibility to arsenic	Predicted	20707922
PURG	Arsenic	increased expression of PURG mRNA	Predicted	15761015
RB1	Arsenic	decreased expression of RB1 proteins	Imprinted	20953137
		decreased phosphorylation of RB1 proteins	Imprinted	12480548
	Cadmium	promotes phosphorlyation of RB1 protein, binding of E2F1 protein	Imprinted	20839231
		decreased expression of RB1 mRNA	Imprinted	20839231
SLC22A18	Arsenic	decreased expression of SLC22A18 mRNA	Imprinted	15725085
SLC22A2	Mercury	decreased activity of SLC22A2 protein	Predicted	17287197
SLC26A10	Arsenic	decreased expression of SLC26A10 mRNA	Predicted	22521957
SLC4A2	Arsenic	increased expression of SLC4A2 protein	Predicted	17018029
SPON2	Arsenic	decreased expression of SPON2 mRNA	Predicted	19128835
	Cadmium	decreased expression of SPON2 mRNA	Predicted	21457566
TIGD1	Arsenic	affects the expression of TIGD1 mRNA	Predicted	18414638
TP73	Arsenic	increased expression of TP73 protein alternative form	Imprinted	16467208<br>15031205
		increased TP73 protein binding to TP53AIP1 promoter	Predicted	15031205
		increased acetylation of TP73 protein	Predicted	15031205
		increased expression of TP73 mRNA	Predicted	19763917<br>20458559
UBE3A	Arsenic	increased expression of UBE3A mRNA	Imprinted	20458559
WT1	Arsenic	decreased expression of WT1 mRNA	Predicted	16966277<br>20471514
		decreased expression of WT1 protein	Predicted	16966277

© 2014 by the authors; licensee MDPI, Basel, Switzerland. This article is an open access article distributed under the terms and conditions of the Creative Commons Attribution license (http://creativecommons.org/licenses/by/3.0/).
